# The ‘Dog Doctors’ of Edwardian London: Elite Canine Veterinary Care in the Early Twentieth Century

**DOI:** 10.1093/shm/hkz049

**Published:** 2019-06-14

**Authors:** Alison Skipper

**Keywords:** veterinary history, specialisation, dog breeding, pedigree, distemper

## Abstract

This article offers the first historical account of Edwardian London’s elite canine veterinarians. Previous historiography identifies increasing veterinary interest in dogs as a mid-twentieth century phenomenon. Despite tension with the mainstream profession, however, an earlier group of specialist veterinarians provided sophisticated canine medical care to London society. Their activities included the policing and investigation of two key threats to the fashionable and lucrative ‘dog fancy’: the devastating infectious disease distemper and the issue of ‘faking’ (show ring cosmetic fraud). This prestigious work gave the canine veterinarians a competitive advantage over their various rivals and enabled the dog fancy to combat the unintended consequences of its own practices on the canine body. This article consequently reveals an early instance of veterinary specialisation, co-driven by client demands and professional politics, and foregrounds the importance of canine biology in the social history of pedigree dog breeding.

In Edwardian London, a small group of highly skilled veterinarians provided sophisticated and innovative treatment for the dogs of the wealthy. Many of their clientele were involved in the fashionable and lucrative world of pedigree dog breeding and showing. Participants in the dog fancy, as it was known, placed a high financial and emotional value on their animals, so were highly motivated to choose the best treatment for them. In a competitive and unregulated marketplace, the pioneering canine veterinarians positioned themselves as elite providers of health care through their well-publicised involvement in the fancy, which elevated their authority and prestige among breeders, but simultaneously alienated them from their professional colleagues.

Despite their high profile at the time, these elite canine veterinarians seldom appear in histories of veterinary medicine or dog breeding. This article aims to rectify this omission by demonstrating their significance to both activities. As I will show, their work provides an early example of veterinary specialisation, shaped by client demands, professional politics and self-interest. Their historical investigation reveals the flourishing of expertise in small animal practice 50 years before its official recognition as an independent professional field. It also provides a novel perspective on the history of dog breeding, which has generally foregrounded the cultural interpretation of the canine body and thus often overlooked its biological vulnerabilities.

Most previous literature on veterinary history has addressed veterinary politics, education and livestock diseases of economic or public health significance.^1^ Dogs have mainly featured in this historiography in relation to virology or public health.^2^ The few accounts to consider companion animal practice have predominantly focussed on its post-World War II development, which they have generally attributed to increasing prosperity and urbanisation.^3^ Analysing the negotiation of pet care between owners and clinicians, they have little to say about the professional politics of specialisation.

Recent key work by Andrew Gardiner has taken a different approach, which addresses the politics of this ‘small animal turn’ in British veterinary practice and relocates its origins to the interwar period.^4^ He shows how, at a time when most male veterinarians directed their attention to horses and farm livestock, animal charities established clinics, staffed by internally trained unqualified practitioners, to satisfy an unmet need for affordable medical care for pets. This professional encroachment, coupled with the concomitant tendency, described by Julie Hipperson, for the first women veterinarians to take up the lower status and more ‘feminine’ work of small animal practice, eventually pushed the reactionary veterinary establishment to claim dogs and, to a lesser extent, cats, as desirable patients, in a paradigm shift confirmed by the launch of the British Small Animal Veterinary Association in 1957.^5^ Summing up this shift, Gardiner remarks, ‘[a]t the beginning of the twentieth century, the term “dog doctor” was considered a professional insult; by the end, this branch of veterinary medicine was the prestigious norm.’^6^

These ‘dog doctors’, London’s early specialist canine veterinarians, are mentioned in a new account of the late Victorian ‘invention’ of the pedigree dog, by Michael Worboys, Julie-Marie Strange and Neil Pemberton.^7^ However, their brief discussion of canine medicine foregrounded fanciers’ attitudes rather than the role of veterinarians, even when discussing distemper, a serious infectious disease often transmitted between dogs at shows.^8^ The profession’s internal historiography and the wider animal history literature have also generally overlooked the early exponents of canine practice.^9^ In focussing upon their practices, identities and relationships, this article reveals a previously unexplored early instance of veterinary specialisation whose emergence was closely linked to the rise of pedigree dog breeding.

The early dog fancy itself, in contrast, has received significant previous historical attention, stimulated by the seminal work of Harriet Ritvo, over 30 years ago.^10^ Authors have explored how breeders socially constructed, physically reshaped and categorised dogs into distinct breeds and have situated these activities within the wider Victorian social context of class, trade and imperialism.^11^ These historians have emphasised the importance of social class and aspiration to early dog fanciers, many of whom were either of high rank or eager to associate with those who were.^12^ However, they have rarely considered actors in the fancy’s extensive supportive infrastructure, such as veterinarians or kennel staff. Moreover, while these accounts often mention fanciers’ discussions of breeding practices or the changing canine body, little is known about the ways in which canine disease and anatomy constrained their efforts to breed and show pedigree dogs. Yet these practical factors both limited the activities of breeders and also drove the establishment of canine medicine as a distinct veterinary specialism. Therefore, an examination of the elite canine veterinarians’ engagement with the dog fancy both illuminates the history of the veterinary profession and demonstrates the significance of canine biology to the social history of pedigree dog breeding.

I begin by identifying the elite canine veterinarians and describing their working practices. I situate them within the wider canine healthcare marketplace and describe the networks that connected them to the dog fancy, showing that their authority among fanciers largely arose from, and was perpetuated by, their response to two problems that particularly threatened dog shows: distemper, then a major cause of canine mortality, and the ‘faking’ of physical attributes to improve a dog’s appearance for the show ring. I also consider how their relationships with the wider profession constrained their other activities and their professional legacy. In these ways, I show how canine biology limited fanciers’ ambitions and how, through their particular engagement with these limitations, early specialist veterinarians both elevated the standards of canine health care and cemented their own reputation as experts.

This article covers the period from the early 1890s to 1914 and draws on two extensive bodies of primary literature: the weekly veterinary press, consisting of the *Veterinary Record* and the *Veterinary News*, which was the main vehicle for the debate and dissemination of news, gossip and technical information within the profession, and the weekly and monthly dog press, which fulfilled the same role for the dog fancy. The latter comprised a variety of titles, including the official Kennel Club journal, the *Kennel Gazette*, and several independent papers. The elite canine veterinarians were prominent actors in both. I have also used archival sources, veterinary textbooks and memoirs.

## The Early Canine Specialists: Who Were They?

**Fig. 1:**
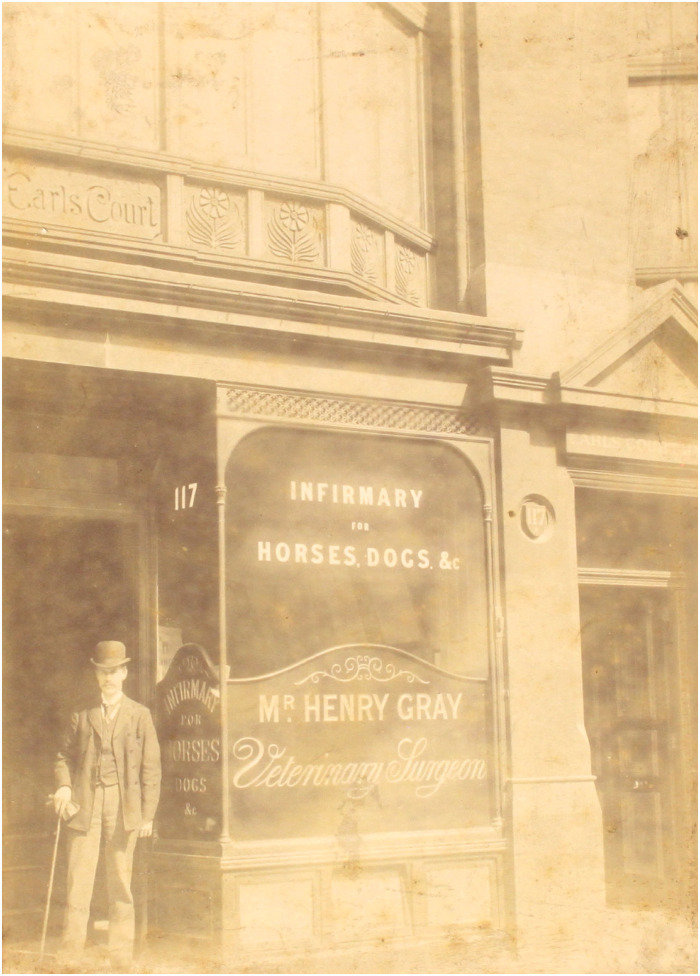
Photograph of Henry Gray’s Kensington practice, c. 1905, photographer unknown. HG/2/2, Henry Gray Papers. Image reproduced with the permission of the Royal Collage of Veterinary Surgeons Trust (RCVS Knowledge)

In 1900, there were 3,417 qualified veterinarians in Britain and over 100 veterinary practices in London.^13^ Most London practices dealt chiefly with horses, but about half-a-dozen, mostly in the West End, were also—or entirely—known for their dog work. Their clientele mostly came from the world of wealthy Edwardian society, where dog breeding and showing was then at its fashionable zenith. In these ‘doggy’ circles, ‘the greatest expert in the canine world’ was Alfred Sewell, ‘“canine surgeon” to the [royal] Household’, who came from a long veterinary dynasty and made an excellent living from his pet-only practice in Belgravia.^14^ Frederick Hobday, also a highly skilled equine surgeon and a professor at the Royal Veterinary College (RVC), ran a practice in Kensington, next door to Henry Gray, who had a particular interest in ophthalmology (and cage birds) (Figure 1).^15^ In South Molton Street, the elderly Charles Rotherham, Queen Victoria’s canine veterinarian, had a keen young partner, E. Lionel Stroud, editor of the low-budget and anti-establishment *Veterinary News*.^16^ A few suburban and provincial veterinarians, such as Ambrose Cornish-Bowden, who practised in Beckenham, and G.H. Livesey, of Brighton, were also closely involved with the dog world.^17^ In addition, high-profile men within the mainstream profession—for example, William Hunting, editor of the *Veterinary Record*, the weekly journal of the veterinary establishment—were sometimes involved with canine matters such as distemper.^18^

In creating a new specialism from canine practice, these veterinarians consciously echoed the contemperanous trend in human medicine.^19^ ‘The great thing now-a-days is specialism, the medical profession carrying this to a fine point … It is the same with domesticated animals’, wrote Sewell, in 1898.^20^ Like those doctors who deliberately cultivated wealthy patients in London or spa towns, they carefully positioned themselves to gain the confidence of affluent owners who could afford costly treatment.^21^ But their interest in dogs was not only mercenary. Sewell, at least, was genuinely ‘fond of dogs’; he ‘much preferred a canine patient to an equine one’, found canine practice ‘much more interesting’ and ‘never regretted’ his decision to abandon horse work and confine himself to the ‘noble and faithful’ dog (and the ‘less faithful’ cat).^22^

The three canine veterinarians who featured most consistently in both the veterinary and fanciers’ press were Sewell, Hobday and Gray. All were recognised by both communities as innovative and highly skilled clinicians. All corresponded in the veterinary journals on canine matters.^23^ All contributed to textbooks largely or entirely about dogs.^24^ All published papers reviewing their canine clinical experiences.^25^ All were frequently praised by breeders for their attention to particular cases.^26^ They offered sophisticated and innovative clinical treatment, including abdominal surgery (such as successful elective Caesarian sections and the removal of bladder stones), pioneering use of anaesthesia and radiography, the invention of new surgical tools and the fitting of prosthetic limbs, all of which were exceptional at the time.^27^ Here, I focus on their public-facing relationships with the dog fancy, so shall not discuss individual patient care further.

These specialist clinicians were well aware of health issues linked to body shape in some breeds of pedigree dog.^28^ For example, Gray described corneal disease in ‘pet dogs that have prominent eyes, such as the pug’ and recognised the predisposition of ‘long backed dogs with short legs, [such] as the Dachshund’ to spinal disease and paralysis.^29^ Hobday noted the increased risk of anaesthetising ‘those breeds (such as pugs, bull-dogs, Blenheim spaniels, etc.) where the nasal bones are short and depressed’.^30^ Sewell observed that Bulldog bitches struggled to give birth ‘due to the modern dogs having such larger [sic] heads’.^31^ All of these problems are still recognised today.^32^ These pioneering descriptions show both that aesthetic modifications to the canine body, driven by efforts to follow a socially constructed ‘breed standard’, affected dogs’ health over a century ago and also that these early canine veterinarians constructed these conditions similarly to modern clinicians.^33^ Unlike modern veterinarians (and some Edwardian fanciers), however, they made no public criticism of these breeding practices. As discussed later, they may have been too preoccupied with tackling distemper, which then killed, in Gray’s words, ‘fully one-fourth of the canine race’, to prioritise conformation-related disease.^34^

## The Canine Healthcare Marketplace

While the elite canine veterinarians were highly knowledgeable about dogs, most of their colleagues were not. Lagging behind regulatory legislation for other healthcare professions, such as the 1858 Medical Act, the 1881 Veterinary Surgeons Act had recently restricted the term ‘veterinary surgeon’ to practitioners who had formal training from a veterinary school and were registered with the Royal College of Veterinary Surgeons (RCVS).^35^ The mainstream profession was subsequently eager to differentiate veterinarians from unqualified ‘quacks’ by reforming the equine-focussed veterinary curriculum to introduce economically important and ‘scientific’ subjects relevant to agriculture and public health, then central to veterinary ambition and professional identity.^36^ The dog was irrelevant to this agenda. Gray complained in 1904 that ‘canine practice … was not taught at all, or, if taught, was taught in a very perfunctory manner’.^37^ Breeders were largely justified in believing that a ‘lack of knowledge regarding the ailments of the canine race [was] rampant in the veterinary profession’.^38^ Yet some grass roots veterinarians did treat dogs and strove to improve their canine knowledge. Provincial practitioners across the country submitted canine case reports to veterinary journals, and local veterinary meetings quite often featured discussions of canine work, sometimes led by visiting canine specialists.^39^ Some general veterinarians used the elite canine specialists as a referral service, requesting written advice or dispatching difficult cases for a second opinion.^40^ However, while these vets might treat dogs alongside horses and livestock, they had little involvement with, or interest in, the affairs of the dog world.

Many veterinarians, indeed, were not only ignorant of canine medicine but also regarded it as ‘beneath [their] notice’.^41^ One practitioner who began his training in 1909 recalled ‘that we should so demean ourselves as to treat pets as a principal means of livelihood was unthinkable’.^42^ These veterinarians regarded dog work as unmanly, because of its emotional and feminine connotations—as evoked by the despised ‘dog doctor’ epithet. As one wrote: ‘If he [the dog vet] … is content to be spoken of as a “dog doctor” … he will need as much patience as a dressmaker or milliner with the sentimental owners.’^43^ Even Gray, a canine specialist, considered that ‘[d]ogs and cats should be treated as luxuries’ and that owners willing to pay ‘from 10 to 1000 times the worth of a favourite animal’ for successful treatment were ‘cranks and hysterics’.^44^ Some show dogs were extremely valuable and for that reason were considered more worthwhile patients.^45^ However, many veterinarians considered the fancy breeds generally inferior to working dogs, deeming them inbred and useless; one wondered why anyone would ‘breed … neurotic pets, from closely related prize animals, with points of no value outside the immoral ring of dog fanciers’.^46^ These veterinarians had little desire to engage with the dog fancy.

In London, the main competition to the elite canine veterinarians thus came not from their professional colleagues but from unqualified quacks and medical doctors. Despite the restrictions of the 1881 Veterinary Surgeons Act, veterinarians did not yet have a monopoly on treating animals.^47^ Much dog and cat treatment was provided by unqualified practitioners, who, because of the Act, often used terms such as ‘dog doctor’ to describe themselves—another reason why the term was so disliked by the veterinary establishment.^48^ While most veterinarians felt that ‘[a] canine and feline medical expert who is not a veterinary surgeon is also a fraud’, few outside the profession held this protectionist view; as in the human medical marketplace, quacks could practice freely, provided they made no attempt to falsely claim veterinary qualifications.^49^ The most successful quacks provided canine veterinarians with significant competition, facilitated because they, unlike qualified veterinarians, were unfettered by restrictions on advertising and self-promotion, imposed and enforced by the RCVS, the profession’s governing body. The best known was Mr Musgrave, a large-scale Borzoi breeder, deeply involved in the dog fancy, who brazenly advertised himself as ‘Not a Vet., but a Practical Man’. He and his wife ran a flourishing business in Putney, combining the sale of medicines, a canine sanatorium, a boarding kennel and an export service for dogs (and medicines) to India, besides providing medical advice by post at half the price of a vet.^50^ But, for all his success, he did not have the formal authority of a qualified veterinarian and was not part of their coterie.

Medical doctors, unlike quacks, rarely aimed to make a living from animals, but often were interested in their diseases, and sometimes treated them as patients.^51^ Owners might opportunistically ask visiting doctors to examine pets, which vets generally regarded as professional encroachment. ‘It is more than vexatious when an owner … remarks … “my doctor … examined the dog and said you had made a great mistake”’, Hunting complained acidly.^52^ Some high-profile canine enthusiasts were themselves doctors and thus already embedded in medical professional networks.^53^ For this reason, and because of the perception that ‘few vets know much about dogs’, fanciers sometimes asked doctors to attend difficult or prestigious canine cases, as when three doctors, including two ophthalmic specialists, were called to (unsuccessfully) treat the injured eye of a top coursing greyhound.^54^ Veterinarians complained that doctors regarded them as ‘men of a lower social status’ and were prone to an ‘offensive tone of superiority’ towards them, a prejudice reinforced by the unavoidable fact that equine veterinarians had to visit stables, so often dealt with coachmen rather than their employers.^55^ The elite canine veterinarians, with their West End surgeries, therefore deliberately positioned themselves as socially acceptable and highly skilled alternatives to doctors, equally welcome in society drawing rooms, in order to compete for high-end canine work. This polished image enabled them to charge top fees. Gray suggested consultation fees of half a guinea ‘in wealthy districts’, or a guinea for expert work, whereas many practitioners only charged ‘1s. up to 5s. … for examination and advice’; medical fees for comparable services ranged from 2s/6d to 10s/6d at this time.^56^ Like society doctors, they dressed to demonstrate their ‘genteel status’.^57^ Other veterinarians, decades later, recalled Sewell’s ‘pompous and frock-coated’ persona, Hobday’s penchant for ‘spectacular showmanship’ and Stroud’s ‘attire … of a Harley Street physician’.^58^ When Sewell, calling at an owner’s front door, was asked to come to the servants’ entrance next time, he ‘refused to examine her dog, and politely added she evidently wanted to see a kennelman’.^59^ On such distinctions were professional reputations maintained.

## The Elite Canine Veterinarians and the Networks of the Dog Fancy

Located in the most affluent part of London, and charging high prices, the elite canine clinicians largely served the wealthiest dog owners and, like society physicians of the time, boasted of doing so.^60^ They used the ready-made networks of Society’s canine enthusiasts to build their clientele. Both Sewell’s and Rotherham’s practices flaunted their Royal Warrants, which naturally recommended them to aspirational fanciers.^61^ ‘Our clients, of course, come from the higher classes’, another canine vet remarked in an interview for the ‘doggy’ press. ‘I happened to cure a favourite dog of Lord Marcus Beresford’s, and that casual introduction brought me twenty good clients in a week.’^62^ It is impossible to know to what extent such statements were themselves intended as advertisements to a readership of potential clients, or what proportion of their clientele were actually aristocrats. Yet most of the elite veterinarians engaged with various forms of self-promotion, treading a fine line between effective publicity and breaching professional advertising restrictions. Sewell was the boldest. He dropped hints about his royal connections in interviews and articles in the dog press, revealing that Princess Alexandra’s spaniel was ‘a sweet tempered little creature’ and describing glamorous trips to the Continent to examine the pets of expatriate nobility.^63^ His rare social appearances strategically revealed his exalted connections, as when his Bulldog participated in charity collection alongside a Dachshund belonging to the wife of Herbert Allingham, surgeon to the Prince of Wales.^64^ For years he wrote educational articles and an advice column (promoting his own obstetric and clinical expertise) for the dog press, with a postal consultation service that included post-mortem examinations.^65^ He held the contract for a quarantine kennel and canine sanatorium in Croydon, owned by Spratt’s, a patent medicine and dog food firm, who used his name in their advertisements.^66^ Such activities not only continually brought Sewell and, to a lesser extent, the other canine specialists, to the notice of the dog fancy, but also annoyed and alienated their professional colleagues, as discussed later.

The recognition of these veterinarians as canine experts was reinforced by their close social links to the dog world. Sewell was Official Veterinary Surgeon to the Kennel Club (KC), the regulatory body of the dog fancy (and a private club, all-male until 1974).^67^ As discussed later, this directly brought him much expert witness work. Many of the canine specialists were fanciers themselves. For example, Sewell kept Bulldogs and, with Hobday, served on the committee of the Bulldog Club.^68^ Cornish-Bowden, himself a serious breeder, was deeply involved with the KC, serving on its General Committee.^69^ In addition, there was the covert bond of Freemasonry. The KC was so intertwined with Masonry that it opened its own lodge in 1907.^70^ Before that, however, several of its most influential leaders belonged to the Imperial Lodge, as did Sewell.^71^ Hobday, Stroud and Cornish-Bowden were also Freemasons.^72^ Freemasonry linked the leaders of the KC with the leaders of wider society. Edward VII, the common patron of Sewell and the KC, was a well-known Mason, as was the Chief Commissioner of Police, Sir Charles Warren, who followed Sewell’s suggestion in using bloodhounds (provided by a KC member) in the hunt for Jack the Ripper.^73^ These influential networks may well explain Sewell’s apparent immunity to disciplinary action from the RCVS, which normally cautioned or expelled those who blatantly flouted its regulations on advertising, as he did. Gray, however, who was neither (apparently) a Mason nor involved in the fancy, secured his position among the elite canine veterinarians more through his contributions to veterinary knowledge, which I will discuss later, than by a flamboyant public presence, like Sewell or Hobday.

While informal social networks reinforced the close professional relationships of the elite canine veterinarians with the dog fancy, their public engagement with it centred, to mutual advantage, on their involvement with two major problems of the canine body which, in very different ways, threatened show culture. Both through their work in averting the threat of distemper and their role in the detection of faking, the elite canine veterinarians defended the practices of the fancy and gained status from the authority thus conferred on them, in a mutually beneficial symbiosis.

## Distemper

Among all the gossip and competition of the Edwardian dog fancy, the fear of distemper united every faction. As dog shows had become larger and more elaborate, so too had distemper become an increasing problem. ‘[V]ery few dogs reach [ten years] without being attacked’, Sewell noted.^74^ The symptoms were well known: infected dogs became ill and feverish, might develop pustules on their skin, coughing, discharging eyes or diarrhoea and, after days or weeks of illness, might slowly recover, perhaps with permanent ill-effects, or, very often, would die.^75^ Although the canine experts regularly published and discussed their treatments for distemper, all agreed that, while good nursing might help a dog recover, no medication made much, if any, difference to the outcome.

While every dog owner feared distemper, the problem was particularly important within the fancy, for many reasons. Distemper was recognised as an infectious disease, consequently often transmitted between dogs at shows.^76^ Pedigree dogs were widely thought to suffer more severely, because of inbreeding, with ‘foreign breeds’ and short-nosed dogs particularly vulnerable.^77^ The high mortality had obvious financial implications. In 1904, a particularly bad year, Hobday noted that ‘[h]undreds and hundreds of pounds worth of dogs had been lost in single kennels alone this year from distemper.’^78^ Such disaster exerted a significant emotional toll on owners: ‘[w]hat harrowing tales of woe and distemper follow in the wake of a Dog Show’, wrote one leading breeder.^79^ The problem was so great that some fanciers refused to show their dogs at all. Even Sewell admitted that his wife’s Japanese Spaniel ‘is never allowed to go out except into the back garden, for we are so afraid she might catch that beastly distemper, and die’.^80^

**Fig. 2:**
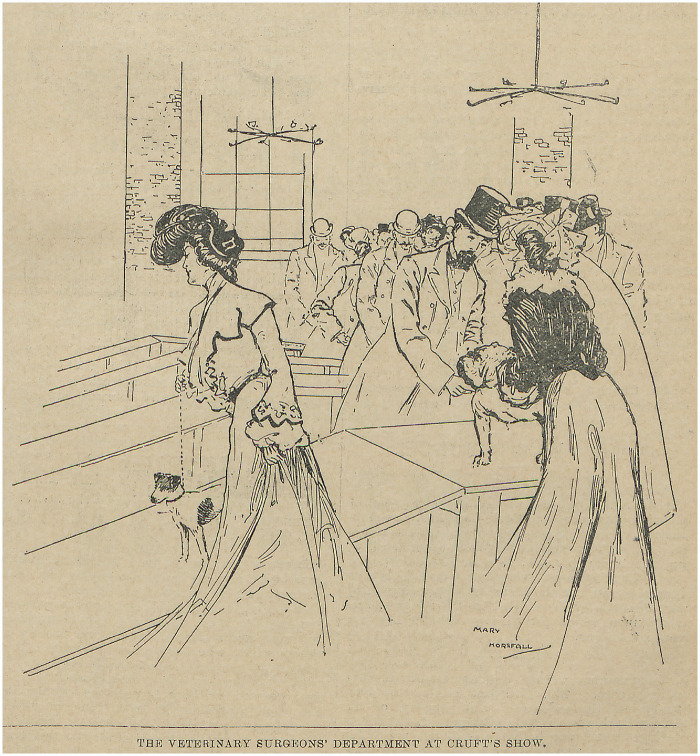
‘Vetting-in’: the veterinarian in the foreground, wearing a top hat, is Alfred Sewell. Mary Horsfall, ‘The Veterinary Surgeons’ Department at Cruft’s Show’, *The Illustrated Kennel News*, 13 February 1903, p. 147. © British Library Board, Lou.Lon.907 [1903], used with permission.

Most fanciers, however, were simply not willing to stop exhibiting their dogs. Attention therefore turned to reducing the chance of dogs contracting distemper at shows. Worboys *et al.* have described early efforts to enforce disinfection of show benching, on which dogs were exhibited, following lobbying by the prominent breeder Everard Millais.^81^ Although its effectiveness was disputed and its implementation patchy, such measures became compulsory.^82^ In parallel, veterinarians were appointed to examine every dog for signs of distemper before its admittance to each show. At larger shows, this work was almost always carried out by the elite canine veterinarians (Figure 2). It was a major logistical enterprise, because so many dogs were involved, many arriving by train, and some staying overnight at the show. One sycophantic account described the process in 1901:

An important event like the Kennel Club Show begins really the evening before the opening … [T]he dogs … begin to arrive fast and in clusters. They are received by Mr. Alfred Sewell, MRCVS, and his partner, Mr FW Cousens, MRCVS, who stick to their post till late into the night, carefully examining each exhibit, sending those unfit for examination at once into the commodious hospital, and putting doubtful cases aside for closer scrutiny when there is a temporary lull in the arrival. The temperature of … the dogs whose appearance makes such a precaution necessary is tested with the clinical thermometer. When the last train has arrived the work is finished for the time being.^83^

This show, held at the Crystal Palace, involved almost 1,500 dogs: Sewell and Cousens would have returned the following morning to examine the second cohort of arrivals. Yet veterinarians were not paid for this gruelling work, although they might, for instance, receive silver cigarette cases instead.^84^ There were even rumours, according to the gossiping *Veterinary News*, that ‘[s]ome … thought the advertisement of being veterinary surgeon to a big dog show worth £50’.^85^ ‘Vetting-in’ was an unparalleled way to appear before large numbers of potential clients in a position of authority: small wonder that the elite canine veterinarians were keen to participate and perhaps even pay for the privilege.

It was generally acknowledged, however, that the system was fraught with difficulties. The sheer number of dogs that each vet was expected to examine was problematic. ‘It is an impossibility for one man to examine say two hundred dogs in a couple of hours and do himself and his profession justice’, Livesey, a regular show vet, complained.^86^ Moreover, while the elite canine veterinarians generally commanded respect (Sewell—secure in his authority—was considered particularly strict with admissions), veterinarians with less canine expertise often officiated at provincial shows. These practitioners might be careless, ‘many exhibits never coming under the observation of the veterinary inspector at all, whilst the remainder are passed over quite casually’, or cowed by a conflict of interest when examining dogs ‘which belonged to their own clients’.^87^ Inevitably, therefore, exhibitors often challenged veterinary decisions, complaining that their own exhibits had been unjustly excluded or their rivals’ dogs incompetently passed, even though clearly ill.^88^ These bitter disputes might end in official hearings, supported by contradictory certificates, which frequently enlisted the superior opinions of one or more elite canine veterinarians to confirm or contradict the judgement of less respected colleagues.

Furthermore, in spite of vetting-in, dogs still caught distemper at shows. Even Sewell admitted, with ‘over twenty years’ experience, that the examination of dogs at Shows … has not prevented dogs contracting distemper at these places’.^89^ He noted that ‘[t]he Veterinary Surgeon is often blamed’ for transmitting infection between dogs and admitted that ‘this can easily be done if he is not careful’. Sewell described his own precautions; he would ‘rinse [his] hands in some strong disinfectant’ if ‘examin[ing] a dog who shows the least suspicion of … contagious disease’.^90^ He countered suggested reforms for a purely visual examination (which, he argued, would be inadequate) and for disinfection after every dog (which would be too time-consuming). His view was that he and the other elite canine vets provided a useful defence, however imperfect, against the spread of distemper, rather than causing it. His colleagues unanimously agreed. ‘[N]ever have [I] seen the slightest laxity, or the passing of any dog suffering from suspicious symptoms’, Stroud wrote, defending officiating veterinarians *en bloc* in spite of a personal hatred for Sewell.^91^ By presenting a united front on the importance of vetting-in, the elite canine vets reinforced their own unique communal authority within the fancy.

The elite canine vets similarly worked together to negotiate the technicalities and politics of vetting-in with the KC and local dog show societies. In 1912, Cornish-Bowden acted as spokesman for Sewell, his partner Frederick Cousens, Hobday and Gray to challenge a KC regulation, which only allowed inspecting veterinarians to exclude an exhibit if they could diagnose ‘a specified infectious or contagious disease’.^92^ This edict, he complained, meant that dogs in the early stages of distemper were passed into shows because the veterinary officials could not be certain of their diagnosis. The assembled committee were incredulous; ‘Surely a veterinary surgeon should be able to say whether a dog is suffering from a contagious disease?’ Cornish-Bowden’s explanation of clinical uncertainty only convinced them because he enlisted the united authority of the canine specialists; ‘the veterinary surgeons we got the views of, all admit they cannot’.^93^ But every such negotiation was hard-fought. During this discussion, recommendations to limit the number of dogs each vet was expected to examine, and to stop using unqualified practitioners as substitutes, were only passed by a narrow margin, and the suggestion of paying show vets was immediately quashed.^94^ Similarly, although the elite canine vets frequently complained that it was ‘hardly fair’ to ‘vet-in’ ‘without fee or reward’ at large dog shows where quack medicine vendors, their direct competitors, were allowed ‘to solicit orders for their nostrums’, they rarely persuaded show committees to exclude them.^95^ Fanciers wanted to buy quack medicines, which were cheaper than consulting a vet, and show committees depended on the merchants’ commercial revenue. Veterinarians had little choice but to accept the situation: shows were simply too important a showcase for their specialist expertise for them to do otherwise.

Vetting-in was a makeshift control measure for distemper in the absence of any effective vaccine or treatment: both fanciers and canine veterinarians were keenly interested in research into the disease. Both obviously wanted to improve canine health; veterinarians also hoped that effective intervention would, as the hallmark of the qualified professional, differentiate them from less knowledgeable rivals. From the 1890s, research centred on two linked problems: identifying the causal organism of distemper and developing an effective vaccine. Around 1900, most experts believed distemper was caused by a bacterium, although opinions differed on which.^96^ Doctors, scientists and veterinarians throughout Europe and beyond, in institutions and clinical practice, strove to create attenuated vaccines from these putative causal bacteria.^97^ The canine veterinarians keenly debated these innovations and enlisted breeders as participants in practical tests of new vaccines.

In 1900, there was a surge in distemper research. Sewell and the eminent pathologist John McFadyean, dually trained as a veterinarian and a doctor, who was then working at the Jenner Institute, ‘offered their services gratis’ in a collaborative effort to develop a vaccine, bankrolled by wealthy fanciers such as the Duchess of Newcastle.^98^ Meanwhile, Sidney Copeman, a medical pathologist working at the Lister Institute, suddenly launched his own vaccine: so, in 1901, did Dr Phisalix of the Natural History Museum in Paris, who was strongly championed by Gray.^99^ Previous efforts to produce a distemper vaccine had, as in contemporary agricultural initiatives, involved empirical inoculation practices extrapolated from smallpox control, such as ‘lymph inoculating threads’ for insertion under the skin, made and sold privately by an older canine veterinarian.^100^ Sewell’s approach to the new products was informed by prior experience with these earlier vaccines, while Gray had an entirely different expectation of vaccination, rooted in laboratory science, particularly the new discipline of bacteriology. This difference developed into an impassioned dispute in the *Veterinary Record* between Sewell and Gray, echoed and discussed further in the dog press.

**Fig. 3:**
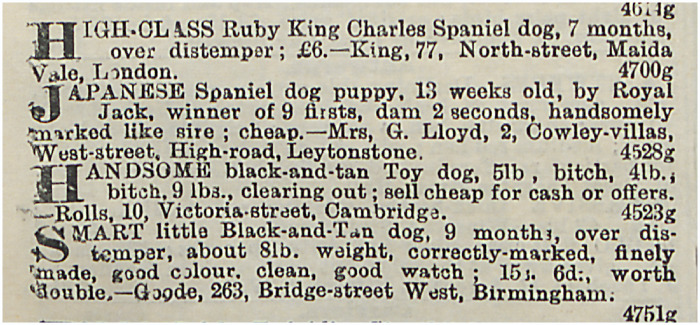
Advertisement column mentioning dogs ‘over distemper’. ‘Toys - Spaniels and Terriers’, *The Stock-keeper and Fanciers’ Chronicle*, 7 January 1898, p. xvii © British Library Board, LOU.LON.418 [1898], used with permission.

Sewell grounded his understanding of distemper in his extensive clinical experience. Like most breeders, he believed that dogs that recovered from distemper were subsequently immune. Dealers’ advertisements often listed ‘over distemper’ among dogs’ other desirable attributes: no one wanted to buy an expensive dog that might imminently die (Figure 3).^101^ For Sewell, an effective vaccine would mimic this natural immunity and prevent dogs contracting distemper. He made his stance clear:

I have no faith whatever in dogs having real distemper twice. I have been so convinced of this for years that in the winter when I am not showing my valuable bull dog … I for convenience sake keep him in my distemper hospital, and he has never had a second attack. If dogs may have distemper as often as they come in contact with it then what is the use of vaccinating at all?^102^

As each new vaccine was launched, Sewell therefore requested a sample to test himself, pragmatically vaccinating a group of dogs, introducing a newcomer suffering from distemper, and observing the illness and death which followed. On this practical basis, he soon declared the Copeman and Phisalix vaccines useless.^103^

Gray, in contrast, entered the debate as Dr Phisalix’s agent. He had formed connections with French scientists through translating their veterinary articles for British journals.^104^ Veterinary bacteriology in France—following Pasteur—was then more advanced than in Britain, whose late nineteenth-century veterinarians had instead prioritised practical disease control.^105^ Consequently, through importing the Phisalix vaccine, Gray could position himself as uniquely close to the latest international institutional science, thus enhancing his expert status. He insisted that vaccine users should follow Phisalix’s complicated protocol and give each dog three inoculations; if the dog developed a reactive swelling at the injection site, then vaccination had been effective.^106^ However, while he claimed that successful vaccination increased resistance to distemper, Gray also believed that dogs could contract distemper more than once.^107^ By definition, therefore, he could not guarantee that vaccination would produce immunity to the disease and thus failed to satisfy Sewell’s fundamental requirement for an effective vaccine.

During 1902, the dispute intensified. A second trial of both vaccines, under the direct supervision of Gray and Copeman, also ended in failure.^108^ Gray continued to defend the vaccine, criticising Sewell’s premises, the vaccine batch (‘Living drugs … are all uncertain in their action’) and even the idea of deliberately challenging the vaccine, complaining that Sewell ‘recklessly allowed his dogs to contract disease’.^109^ Sewell enlisted influential dog people to support his position; one Master of Foxhounds complained that ‘my huntsman and I consider … that the use of the [Phisalix] vaccine is *worthless*’.^110^ He thus reinforced his authority among fanciers through their own practical experience. Gray, who in contrast aimed to present himself to a professional audience as a practitioner-scientist, was trying to construct a ‘scientific’ measurement for success but, in Sewell’s eyes, losing sight of the intended goal.

Since this controversy had largely played out in the *Veterinary Record*, William Hunting, its editor, eventually intervened and organised a committee of veterinarians to supervise further trials of the Phisalix vaccine, in a research pattern then also used to investigate livestock disease.^111^ Committee members included Sewell, Gray, Stroud and McFadyean, as well as several mainstream practitioners.^112^ These trials extended throughout 1903, using puppies (and funding) sourced by appeal to both vets and breeders.^113^ Although extensive efforts were made to prevent contagion, the trials were fraught with disaster, as successive batches of puppies inadvertently contracted distemper before they had been fully vaccinated, rendering the work invalid.^114^ Eventually, a majority of the committee decided that ‘the third experiment [provided] unimpeachable evidence that the vaccination failed to confer any immunity against distemper’.^115^ Gray and Stroud, however, disagreed, publishing a ‘Minority Report’, which argued that all the trials were so compromised by infection as to be useless.^116^

In this acrimonious affair, politics and science became inextricably tangled. Veterinary onlookers questioned Gray’s authority, as an ‘obscure suburban practitioner’, to oppose the revered McFadyean.^117^ However, Gray clearly sincerely believed in the vaccine and, in spite of their dispute, maintained a professional relationship with Sewell, even making private jokes in their published correspondence.^118^ Stroud, however, hated Sewell, in an enmity shared with his business partner, Rotherham, whose Royal Warrant had been eclipsed by Edward VII’s preference for Sewell. He regularly used his position as editor of the maverick *Veterinary News* to criticise Sewell.^119^ Stroud also resented the veterinary establishment. He was repeatedly defeated in RCVS council elections, and Hunting considered the *News* ‘a cheap and nasty specimen of journalism’.^120^ Stroud’s support of Gray thus probably reflected his loathing of Sewell and Hunting more than the disinterested pursuit of science.

The intricacies of this specialised distemper debate were not followed by most mainstream practitioners. One veterinarian, bemused by ‘such elaborate precautions as Mr Gray requires’, sarcastically wondered why anyone would

seek to obtain immunity from distemper by rearing pups in a glass case in the middle of a ten-acre field, surrounded by a moat filled with disinfectant, the drawbridge guarded by a chemist whose duty it would be to sterilise all food and water, and fill up his time in bottling sunshine for the benefit of the pups on dull days?^121^

This barbed comment firmly dismissed Gray’s approach to distemper as unrealistically impractical. But its efficacy was also widely questioned: by 1905, Gray’s support of the Phisalix vaccine became an increasingly marginal position, as veterinarians and breeders across the country reported their failures with it.^122^ ‘No greater imposture was ever put before a credulous public’, one breeder bitterly commented.^123^ Some years later, Gray himself ruefully acknowledged that ‘distemper vaccines … prepared from visible microbes … do not … fulfil the claims put forth by their discovers or exploiters.’^124^ Ultimately, he was too conscientious not to revise his beliefs in line with new research. Moreover, like the other canine veterinarians, he knew that his expert knowledge and professional integrity were key to defining and retaining his elite reputation. For the top canine specialists also served as regulatory enforcers to the dog fancy.

## Faking

The artificial manipulation of canine bodies to improve their appearance for the show ring was a constant concern for the KC. At the more extreme end, rumours described ‘sculptors of living canine flesh’, who could ‘by a little surgical manipulation of a puppy … increase its value 200 or 300 per cent’.^125^ Such procedures may have been apocryphal, but less drastic interventions, such as altering the shape of dogs’ ears and trimming or dyeing the coat, were commonplace yet, under KC regulations, absolutely forbidden (with the exception of fancy hairstyles in Poodles). Faking both fraudulently increased the value of particular animals and also undermined the whole pedigree system by giving them physical attributes that could not be inherited. Therefore, it struck at the heart of the fancy. Its importance led the KC to enlist the elite canine veterinarians as enforcement agents.

Infractions of the faking rules were numerous, varied and generally identified through the whistleblowing of rival fanciers, who would report their enemies to the authorities in the hope of seeing them penalised. Faking cases were often first detected at shows. Show vets—who were in attendance anyway, to deal with distemper—were therefore often called to examine suspicious animals immediately. In one typical incident, at a show in 1904, Sewell was asked to examine a Brussels Griffon suspected of a dyed head. Sewell ‘used a handkerchief and rubbed its head …with benzene … a quantity of colouring matter came off’.^126^ The subsequent formal disciplinary hearing involved solicitors on both sides, numerous testimonies from other exhibitors and the appearance of three elite canine vets as expert witnesses—Sewell, Gray and Stroud. Although the hearing lasted all day, Sewell only attended for a few minutes yet charged two guineas to appear.^127^ While he probably commanded the largest fee, this suggests that all the expert witnesses found such work lucrative, besides benefiting from the covert advertising of the surrounding press coverage.

A remarkable amount of effort went into investigating some complaints. If exhibitors distrusted the local veterinarians who officiated at provincial shows, Sewell, as official veterinarian to the KC, might adjudicate. Thus, he was summoned by wire to a Manchester show, to examine an Airedale Terrier which had allegedly been given something (‘I should say Belladonna’, the objector suggested) to artificially dilate its pupils, making its eyes look darker for the show ring.^128^ In 1913, one Scottish Pomeranian exhibitor was accused of dyeing his dog to conceal an undesirable white marking on its chest: two Edinburgh veterinarians could find no trace of dye. KC officials sent the dog to Sewell by train ‘in a locked box’, thus preventing any outside interference: ‘rather suspicious about’ a bald spot on its chest, he kept it and, three weeks later, was vindicated when white hairs began to regrow.^129^ Sewell was particularly punctilious when dealing with these cases. Unlike his colleagues, he took care to note any identifying marks when writing certificates about particular dogs, thus safeguarding himself from fraudulent substitutions, and was inventive in providing practical demonstrations to support his opinion, as when, testifying in another case of suspected eye-tampering, he brought a dog with him to the hearing, one eye pre-treated with belladonna, to demonstrate the drug’s effect.^130^

While ‘faking’ investigations were both common and varied, one particular type of faking had a disproportionate impact within the fancy: the artificial manipulation of dogs’ ears. This was a particular problem in Collies, then one of the most fashionable and expensive breeds. Dealers made huge profits through finding or breeding good specimens in rural Britain and selling them on to wealthy fanciers in London or America.^131^ Among other attributes, a good show Collie required a particular ear shape, generally described as ‘semi-erect’ or ‘tipped’, with most of the ear pricked but the very tips bent over. It was temptingly easy to ‘fake’ the preferred bent tips, either by applying small weights to them for some months, or through surgery, thus vastly increasing the worth of the dog. This practice consequently attracted particular attention within the fancy, and the elite canine veterinarians—particularly Sewell—were key to its regulation, as two examples will show.

**Fig. 4:**
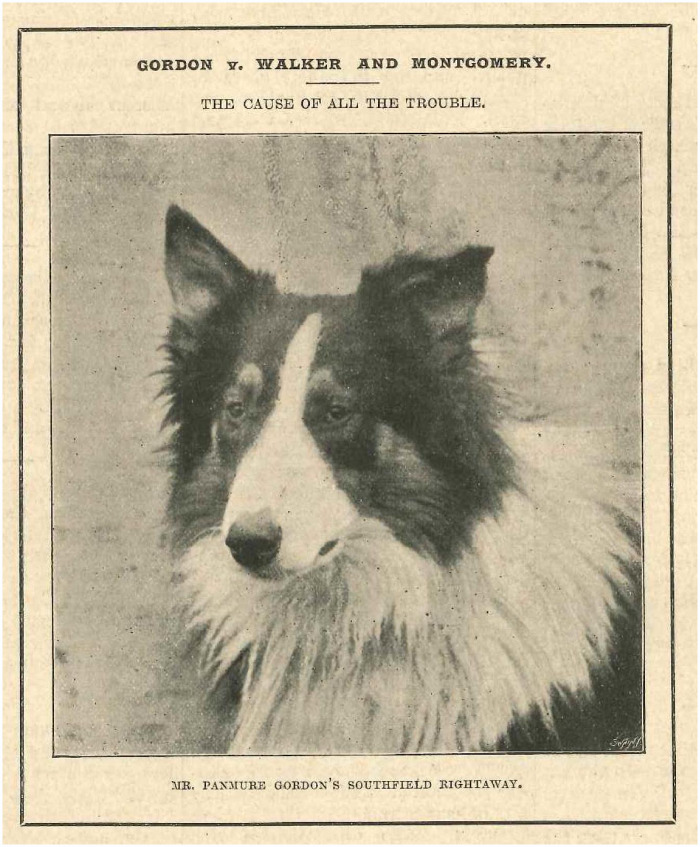
Southfield Rightaway, photographer unknown. *The Kennel Gazette*, February 1899, p.52. Reproduced with permission of the Kennel Club Limited.

The first of these cases concerned a Collie called Southfield Rightaway, the subject of a Scottish legal action in 1898.^132^ Rightaway was bought by an agent for Mr Panmure Gordon, a stockbroker and President of the Scottish Kennel Club, from two working-class fanciers who, like many others, bred and showed dogs in the hope of producing a valuable top winner: the purchase price of £100 represented more than a year’s wages for both men together.^133^ At first, Rightaway’s ears were correctly ‘tipped’, but Gordon soon noticed they were becoming more pricked (Figure 4). He discovered that the vendors had previously attached small gelatine lozenges to the dog’s ears, to ‘tip’ them before his show debut. Asserting that ‘he would not have bought the dog had he known that artificial means had been used’, Gordon took them to court.^134^

Unusually, the facts in this case were not disputed; ‘Walker, one of the defendants, said … all exhibitors weighted the ears of prick-eared dogs.’^135^ Nor was Walker’s character questioned, despite his working-class status: observers remarked he ‘made by far the best witness called on his own side, and … only did what he knew others were doing, and saw no harm in it’.^136^ Everyone acknowledged that weighting ears was widespread in the Collie fancy: the question was whether it counted as ‘faking’. This was a test case, not an attempt to vilify the defendants personally. Even Gordon himself later noted that he ‘bore (and bear) no malice whatever towards either of the defendants’.^137^

Although several Scottish veterinarians also testified in the trial, they were overshadowed by Sewell, whose dramatic arrival was noted in the press:

Mr Alfred Sewell, MRCVS, who had travelled all night from London to Edinburgh … was next called. He produced a stuffed head of a Collie, by which he was able to demonstrate to the Court the position of a properly-carried semi-erect ear and a prick ear … In Mr. Sewell’s opinion … as a veterinary-surgeon … “the manipulation applied to Southfield Rightaway was such as to alter the natural anatomy temporarily.”^138^

Here, Sewell used his favoured approach of providing a practical ‘visual aid’ to illustrate his testimony and explicitly used his professional authority to underline his stance. Although the judge found in favour of the defendants, this was not because he questioned Sewell’s expert status but on the basis that there had been ‘no attempt to deceive’ and that therefore Panmure Gordon should lobby the KC to alter its regulations rather than accuse the vendors of fraud.^139^ Gordon took his advice and was later reported as ‘successful in his self-appointed task’, having achieved an ‘alteration of rule [which] forbids ear manipulation … of any kind’.^140^

Commentators generally thought that ‘Panmure Gordon has done a great deal of good’.^141^ Yet one writer noted that ‘if the agent … had used the caution necessary in buying £100 dogs he would never have bought it without veterinary advice’.^142^ Not only was Sewell the acknowledged pundit on canine anatomy, but, in providing an impartial assessment of whether show dogs were ‘manufactured [or] begotten’, he had been vital in underwriting the value of the canine body, the currency of the dog fancy.^143^ However, despite Sewell’s position as official veterinarian to the KC, he still turned to the other elite canine veterinarians to support his judgements. My second example shows the importance of their co-operation in reinforcing critical expert testimony.

Some 15 years after the Panmure Gordon case, another wealthy Collie breeder, Walter Mason, used the ear ‘faking’ ban to challenge a business rival. As Margaret Derry has described, Mason led a hugely lucrative transatlantic trade in the breeding and export of Collies.^144^ His competitors—and enemies—included a man called H.E. Packwood. Keen to eliminate his rival, in 1913 Mason lodged a complaint against Packwood with the KC, citing 23 assorted counts of discreditable conduct.^145^ While the disciplinary committee dismissed most of the accusations, merely reprimanding Packwood for poor record-keeping, one charge constituted a more serious threat to his reputation: the accusation that he had ‘faked’ the ears of a dog called Billesley Bertie.

At a second hearing, Mason brought five Collie fanciers prepared to swear Bertie’s ears were ‘faked’: Packwood brought Bertie.^146^ All present—including Packwood—agreed that ‘there was a good deal missing out of the ears’, which seemed smaller and narrower than normal.^147^ While Mason’s supporters eagerly assured the committee that the ears had been deliberately trimmed, Packwood argued that Bertie had, as a puppy, suffered badly from mange, for which he had been treated with an extra strong solution of arsenical sheep dip ‘thus causing the ears to fester and parts of the ears to come away’.^148^ ‘I made it strong. Kill or cure, that is what I thought’, he explained.^149^

The case clearly hinged on whether Bertie’s damaged ears were caused by disease (or arsenic) or had been ‘faked’. ‘It seems to me that it is very much a question for expert evidence’, the KC Chairman (himself a doctor) remarked.^150^ Unfortunately, this was August, and both Sewell and Gray, who had been asked to attend, were on holiday.^151^ Instead, therefore, Mason’s team presented Professor Wooldridge from the RVC, a highly respected general clinician, who thought Bertie’s ears bore symmetrical scars, more typical of surgical intervention than natural ulceration.^152^ The committee found in favour of Mason and suspended Packwood from any involvement in showing for 10 years.^153^

The furious Packwood contacted Sewell, who examined Bertie and arranged for four other well-known veterinarians, including Cousens, Hobday and Gray, to do the same.^154^ Armed with letters from all five veterinarians stating that they thought Bertie’s ears were *not* faked, Packwood returned to the KC, who consequently reopened the case.^155^ At this final hearing, Mason called on Wooldridge again, supported by Cornish-Bowden. But Wooldridge admitted that he knew nothing about KC rules; Cornish-Bowden unexpectedly agreed with the opposing side and ‘saw no reason to think’ that the ears had been cut; and Packwood’s five veterinarians, all separately appearing, agreed that they could see no visible scarring, that the damage was probably ‘the result of some disease or accident’ and that nobody wanting to improve a Collie’s ears for the show ring would have done it in this manner anyway.^156^ Faced with this overwhelming evidence, the committee reversed their previous decision and revoked Packwood’s sentence.

This incident showcases the importance of the elite canine vets to the judiciary of the dog world. Packwood incurred ‘serious expense’ in employing five expert witnesses but succeeded in salvaging his business. Sewell knew that augmenting his own expertise with that of his colleagues would add weight to his judgment and increase his client’s chances of success; the KC committee respected the united authority of the canine specialists enough to overturn a previous high-profile decision. This authority ultimately depended on their demonstrably superior understanding of canine medicine. The transcript reveals the meticulous care with which the elite canine vets used detailed clinical observations, informed by their long experience, to support their conclusions. Whatever the social mechanisms with which they promoted themselves among breeders, the heart of their expert status lay in this closely studied and recorded empirical understanding of the canine body. They really did know more about dogs than most other veterinarians and knew that their most valuable currency was this trusted knowledge. Consequently, in spite of their internal rivalries and feuds, they maintained a symbiotic mutual relationship, each relying on the others to reinforce and support this shared and esoteric expertise. They needed to do so, for other veterinarians not only challenged their conclusions while knowing less about dogs, like Wooldridge here, but also resented the various practices that consolidated the elite canine veterinarians’ status in the fancy. I conclude this article by considering the friction between the canine specialists and the mainstream veterinary profession.

## The Elite Veterinarians and the Wider Profession

In 1904, an eccentric fancier, the Hon. Mrs Florence Chetwynd, accused Percy Woodroffe Hill, a provincial canine veterinarian, of substituting a worthless puppy for her valuable pedigree bitch while boarding her dogs one winter.^157^ As usual, the ensuing legal case involved witness appearances by several veterinarians, who debated the physical evidence for the age of the dog concerned. Sewell and Gray, although they were then deep in conflict over the Phisalix vaccine, both vigorously supported Woodroffe Hill, arguing that small dogs, even when adult, might have juvenile features such as too few teeth. They were opposed by JAW Dollar, a bombastic equine veterinarian who claimed he ‘had not heard of [Sewell’s] work or doings’, which was certainly untrue, as both were members of Hunting’s distemper committee at the time.^158^ Hobday agreed with Dollar during the hearing—perhaps swayed by their common expertise in prestigious equine work—but later ‘confess[ed] his mistake’ at a professional meeting, ruefully adding he ‘had not been proud since’ of his ability to age a dog correctly.^159^ As discussed earlier, the elite canine vets had a mutual loyalty in the face of outside threat and shared a genuine desire to advance their canine knowledge, which distinguished them, both in the eyes of the fancy and of each other, from outsiders like Dollar.

However, the separation between the canine veterinarians and the mainstream profession was more than a matter of differing expertise. Many of the activities that which enhanced the status of the elite canine veterinarians within the dog fancy, directly conflicted with the restrictive code of professional conduct imposed by the veterinary community, leading to constant friction between them. This enmity was particularly directed at Sewell, in spite of his ‘real professional ability, which is not doubted by his confrères’.^160^ To some extent, they were probably jealous: he was very wealthy, even keeping a personal Italian chef.^161^ Moreover, his constant appearances in the dog press flouted professional guidelines on advertising and enraged other veterinarians. Advertisers, one typical commentator complained, again echoing parallel concerns in the medical profession, ‘savour too much of the quack themselves’.^162^ Feelings ran particularly high when, in 1909, one penny weekly ran an article entitled ‘Royalty’s Dog Doctor’, which described Sewell as:

one of the greatest of living authorities on the ills to which (dog) flesh is heir … To [his] consulting surgery … comes the world of fashion, with footmen carrying sick dogs of all breeds and sizes … Every year Mr. Sewell makes several visits to St. Petersburg to examine the dogs of the Tsar; and he is frequently running over to Paris and Berlin …^163^

This piece, which could fairly be said to mark Sewell as the first ‘celebrity vet’, went beyond acceptable professional limits. It was instantly reprinted in the veterinary press, whose editors would have been particularly revolted by the ‘Dog Doctor’ headline, with its connotations of mawkish sentiment and quackery.^164^ Sewell was forced to respond, asking the newspaper to confirm that he ‘did not supply the information’, and ‘that almost every particular in the article is incorrect’, in order to avert the disciplinary wrath of the RCVS, who could potentially strip him of membership.^165^ Hobday also had to issue rebuttals after similar appearances in the press.^166^ However useful such accolades might be in recruiting clients, these clinicians knew very well how far they could push the veterinary establishment.

But the Edwardian canine specialists did not only breach their colleagues’ expectations on publicity; they also challenged professional norms in other ways. Many of them championed the training of women for veterinary work. Sewell and Cousens were the first veterinarians to offer formal training schemes for women to qualify as canine nurses; Hobday also strongly supported canine nurse training.^167^ Decades later, Hobday, in his capacity as President of the RVC, would encourage the first few women veterinary students in England.^168^ The mainstream profession wholeheartedly opposed all these innovations. They were equally hostile to the canine veterinarians’ charitable work. Sewell supported the aristocrats’ charity Our Dumb Friends League, giving ‘gratuitous advice … to sick animals belonging to poor people’; Hobday ran a ‘Clinique’ at the RVC for the pets of the poor, which also provided his students with small animal experience.^169^ Other veterinarians regularly complained that these schemes constituted covert advertising and unfair competition.^170^ Eventually, however, as Gardiner and Hipperson have shown, both charity clinics and women did colonise small animal practice, supported by canine veterinarians, such as Hobday and Cornish-Bowden, despite continued establishment resistance.^171^

## Conclusion

This article has provided the first historical account of a significant group of Edwardian veterinarians, who achieved a good income, high social status and interesting professional work through positioning themselves as elite canine specialists, and developing a field generally despised or ignored by their peers. It has revealed that these clinicians were deeply embedded in the dog fancy and derived authority from it. Through the control of distemper and the investigation of ‘faking’, they enforced KC regulations and policed the pedigree show ring against its threats. A competitive and unregulated healthcare marketplace drove them to self-promotion. While this publicity brought them custom, it also flouted professional norms and alienated canine specialists from mainstream veterinarians. This drove them into closer mutual alliance and further encouraged them to satisfy the demands of the patrons who protected and supported them. Consequently, the development of canine medicine as a specialist field was powerfully shaped by the dog fancy.

At the same time, dog fancy practices were shaped by the elite canine veterinarians through the limitations imposed by canine biology. Dog shows spread distemper, triggering the scrutiny of ‘vetting in’ and driving vaccine research. Uncontrollable variations in canine body shapes, breaching show ring preferences, led to ‘faking’, whose detection necessitated veterinary expertise. Individual dogs needed veterinary care for their ailments, which were sometimes related to their breed and shape. In responding to the needs of the fancy through these multiple engagements with canine bodies, the elite canine vets upheld its values, supported its practices and facilitated its efforts to use dogs for commercial and social gain.

The wealthy clients who employed these specialist veterinarians wanted the best available treatment, challenging the veterinary establishment’s view that dogs were ‘unworthy’ patients. The protection their patronage provided enabled the canine specialists to challenge regulatory restrictions on advertising and professional codes on female employment and charitable work. Yet in all these respects, as well as their focus on pets, the innovations of the elite canine veterinarians of the early twentieth century merely foreshadowed the professional practices of the future. In the twenty-first century, the veterinary profession would become mostly female, predominantly concerned with small animals, technologically innovative and far less restricted in its practices. Thus, the legacy of the Edwardian canine specialists, shaped by their particular circumstances, in many ways eventually became the mainstream norm.

## Footnotes

1 For example, Abigail Woods and Stephen Matthews, ‘“Little, If at All, Removed from the Illiterate Farrier or Cow-Leech”: The English Veterinary Surgeon, C.1860–1885, and the Campaign for Veterinary Reform’, *Medical History*, 2010, 54, 29–54; Abigail Woods, ‘From Practical Men to Scientific Experts: British Veterinary Surgeons and the Development of Government Scientific Expertise, C. 1878–1919’, *History of Science*, 2013, 51, 457–80; Abigail Woods *et al.*, *Animals and the Shaping of Modern Medicine: One Health and Its Histories* (London: Palgrave Macmillan, 2018); Keir Waddington, ‘The Science of Cows: Tuberculosis, Research and the State in the United Kingdom, 1890–1914’, *History of Science*, 2001, 39, 355–81; Susan D. Jones, *Death in a Small Package: A Short History of Anthrax* (Baltimore: Johns Hopkins University Press, 2010); Anne Hardy, ‘Pioneers in the Victorian Provinces: Veterinarians, Public Health and the Urban Animal Economy’, *Urban History*, 2002, 29, 372–87.

2 Neil Pemberton and Michael Worboys, *Rabies in Britain: Dogs, Disease and Culture, 1830**–**2000* (Basingstoke: Palgrave Macmillan, 2012); Michael Bresalier and Michael Worboys, ‘“Saving the Lives of Our Dogs”: The Development of Canine Distemper Vaccine in Interwar Britain’, *The British Journal for the History of Science*, 2014, 47, 305–34.

3 Susan D. Jones, ‘Framing Animal Disease: Housecats with Feline Urological Syndrome, Their Owners, and Their Doctors’, *Journal of the History of Medicine and Allied Sciences*, 1997, 52, 202–35; Chris Degeling, ‘Negotiating Value: Comparing Human and Animal Fracture Care in Industrial Societies’, *Science Technology & Human Values*, 2007, 34, 77–101.

4 Andrew Gardiner, ‘The “Dangerous” Women of Animal Welfare: How British Veterinary Medicine Went to the Dogs’, *Social History of Medicine*, 2014, *27*, 466–87.

5 Julie Hipperson, ‘Professional Entrepreneurs: Women Veterinary Surgeons as Small Business Owners in Interwar Britain’, *Social History of Medicine*, 2018, 31, 122–39.

6 Gardiner, ‘“Dangerous” Women’, 467–68.

7 Michael Worboys, Julie-Marie Strange and Neil Pemberton, *The Invention of the Modern Dog: Breed and Blood in Victorian Britain* (Baltimore: Johns Hopkins University Press, 2018), 172–73.

8
*Ibid*., 172–76.

9 For typical examples, see J.W. Brittlebank, ‘Eminent Veterinary Clinicians of the Past’, *Veterinary Record*, 24 March 1945, 57, 129–32; Iain Pattison, *History of the British Veterinary Profession, 1791**–**1948* (London: J.A. Allan, 1984); Kathleen Kete, *The Beast in the Boudoir: Petkeeping in Nineteenth-Century Paris* (Berkeley: University of California Press, 1995); Katherine C. Grier, *Pets in America: A History* (Chapel Hill, NC: University of North Carolina Press, 2006).

10 Harriet Ritvo, *The Animal Estate: The English and Other Creatures in the Victorian Age* (Cambridge, MA: Harvard University Press, 1987).

11 Sarah Cheang, ‘Women, Pets, and Imperialism: The British Pekingese Dog and Nostalgia for Old China’, *The Journal of British Studies*, 2006, 45, 359–87; Aaron Skabelund, ‘Breeding Racism: The Imperial Battlefields of the “German” Shepherd Dog’, *Society & Animals*, 2008, 16, 354–71; Philip Howell, ‘The Dog Fancy at War: Breeds, Breeding, and Britishness, 1914–1918’, *Society and Animals*, 2013, 21, 546–67; Neil Pemberton and Michael Worboys, ‘The Invention of the Basset Hound: Breed, Blood and the Late Victorian Dog Fancy, 1865–1900’, *European Review of History*, 2015, 22, 726–40; Margaret Derry, *Bred for Perfection: Shorthorn Cattle, Collies and Arabian Horses* *since* *1800* (Baltimore: Johns Hopkins University Press, 2003).

12 Worboys, Strange and Pemberton, *Modern Dog*, 15.

13
*RCVS Register*, 1900, 276; Woods and Matthews, ‘Cow-Leech’, 36.

14 ‘Club and Kennel Notes’, *Kennel Gazette*, December 1897, 459; *The Illustrated Kennel News* (hereafter *IKN*), 21 February 1902, 29; ‘Dogs and Their Doctors—Mr A. J. Sewell, MRCVS’, *Ladies**’* *Kennel Journal* (hereafter *LKJ*), 1895, 1, 143–44.

15 Bruce Vivash Jones, *Twentieth-Century Veterinary Lives* (Cirencester: Granville Penn Press, 2012), 101–04, 124–28; 1901 England Census, RG13/21, Folio 132, 1.

16 Vivash Jones, *ibid.*, 243–44.

17
*Ibid*., 129; *Kennel Gazette*, February 1913, 70–75; *RCVS Register*, 1920, 226.

18 William Hunting, ‘The Master of the Quorn’, *Veterinary Record*, 16 August 1902, 15, 81.

19 Anne Digby, *Making a Medical Living**—**Doctors and Patients in the English Market for Medicine, 1720**–**1911* (Cambridge: Cambridge University Press, 1994), 33.

20 Alfred Sewell, ‘Experiences of a Veterinary Surgeon’, *The Stock-Keeper and Fancier’s Chronicle* (hereafter *Stock-keeper)*, 23 December 1898, 582–83, 582.

21 M. Anne Crowther and Marguerite W. Dupree, *Medical Lives in the Age of Surgical Revolution* (Cambridge: Cambridge University Press, 2007), 211.

22 Sewell, ‘Experiences’, 582.

23 Henry Gray and A. J. Sewell, ‘Distemper’, *Veterinary Record*, 4 February 1905, 17, 496; Frederick Hobday, ‘Canine and Feline Surgery’, *Veterinary Record*, 16 January 1904, 16, 458.

24 Two early nineteenth-century veterinarians, Delabere Blaine and William Youatt, had written comprehensive books on dogs. The Edwardian veterinarians revised and extended this literature to reflect subsequent advances. See Worboys *et al.*, *Modern Dog*, 27–28; Edward Mayhew and A. J. Sewell, *Dogs* *and* *Their Management* (London: George Routledge & Co., 1910); Frederick Hobday, *Canine and Feline Surgery* (Chicago: W. T. Keener & Co., 1906); Eugéne Nicholas, tr. Henry Grey, *Veterinary and Comparative Ophthalmology* (London: H and W Brown, 1914).

25 A. J. Sewell, ‘Operation for Stone in the Bitch’, *Veterinary Record*, 4 March 1899, 11, 509–11; Frederick Hobday, ‘Öophorectomy in the Bitch and Cat’, *Veterinary Record*, 8 July 1899, 12, 14–15; Henry Gray, ‘Canine Influenza or What’, *Veterinary Record*, 18 March 1899, 11, 539–40.

26 ‘Whispers of Fancy’, *Stock-keeper*, 22 July 1898, 164; ‘Whispers of Fancy’, *Stock-keeper*, 7 November 1902, 397; A. Croxton-Smith to Henry Gray, 18 September 1913, Henry Gray Papers, RCVS Knowledge.

27 Hobday, *Canine Surgery*, 22, 114, 262, 304, 343.

28 Conformation-related disease in pedigree dogs is a highly controversial subject among veterinarians, breeders and activists today.

29 Henry Gray, ‘Diseases of the Eye’, *Veterinary Record*, 15 February 1908, 20, 570–78, 573; ‘Chronic Ossifying Spinal Pachymeningitis in the Dog’, *Veterinary Record*, 16 July 1904, 17, 51–54, 51.

30 Hobday, *Canine Surgery*, 14.

31 A. J. Sewell, ‘Parturition’, *Kennel Gazette*, October 1895, 247.

32 For an overview of the modern debate, see the Dog Breeding Reform Group’s website, particularly <http://www.dogbreedingreformgroup.uk/press-releases.html>, accessed 19 May 2019.

33 Modern activists often assume these issues are a recent phenomenon, a reasonable presumption, given the ample pictorial evidence that canine body shapes have become more exaggerated over time. However, these descriptions show that such problems are not new.

34 Other explanations include reluctance to criticise the fancy, either from self-interest or adoption of its values: also, animal welfare was itself constructed differently at this time; see Hilda Kean, *Animal Rights: Political and Social Change in Britain Since 1800* (London: Reaktion Books, 1998); Henry Gray, ‘Aspirin and Distemper’, *The Kennel*, 1912, 3, 173–79, 173.

35 See Woods and Matthews, ‘Cow-Leech’, 38, 52, for more on this.

36
*Ibid*.

37 Henry Gray, ‘Meeting, Southern Counties Veterinary Medical Society’, *Veterinary Record*, 22 October 1904, 17, 251–60, 253.

38 ‘Kennel Advice’, *Veterinary Record*, 6 February 1904, 489.

39 H. W. Billinghurst, ‘Gastric Distention in a Dog’, *Veterinary Record*, 4 January 1908, 20, 462; E. Lionel Stroud, ‘Operable Diseases of the Eye and Eyelids of Dogs’, paper read to Lincolnshire Veterinary Medical Association, *Veterinary Record*, 25 July 1908, 21, 70–76.

40 Letter from Allan McDougal to Henry Gray, 31 January 1916, Henry Gray Papers, RCVS Knowledge.

41 Herbert Watkins-Pitchford, ‘Epilepsy in the Dog’, *Veterinary Record*, 20 July 1895, 8, 37–39, 37.

42 Reginald Hancock, *Memoirs of a Veterinary Surgeon* (London: The Country Book Club, 1954), 63.

43 ‘The Old Obadiah’, ‘On Getting a Practice’, *Veterinary Record*, 2 November 1901, 14, 273–76, 275.

44 Henry Gray, ‘Fees, Charges, Etc.’, *Veterinary Record*, 28 June 1902, 14, 791–96, 791–92.

45 ‘Canine Practice’, *Veterinary Record*, 26 February 1916, 28, 385.

46 ‘Cave Canem’, ‘Vaccination of Dogs’, *Veterinary Record*, 6 May 1905, 17, 714.

47 Woods and Matthews, ‘Cow-Leech’, 30.

48 ‘Quack Dog Doctors’, *Veterinary News*, 6 January 1906, 12.

49 G. H. Livesey, ‘Canine and Feline Experts’, *Veterinary Record*, 6 October 1906, 19, 205–07, 206; Digby, *Medical Living*, 57.

50
*LKJ*, 1897, 6, 99; 1899, 9, advertisement opp. 721.

51 For more on medical involvement in animal disease, see Woods *et al.*, *Animals and the Shaping of Modern Medicine*.

52 William Hunting, ‘Sauce for the Goose’, *Veterinary Record*, 30 March 1901, 13, 541–42, 542.

53 For example, the second Chairman of the Kennel Club was a doctor; ‘The Late Mr Sidney J. Turner’, *Kennel Gazette*, February 1920, 26.

54 ‘Argand’, ‘Dr Gordon Stables’ Advice’, *Veterinary Record*, 22 September 1906, 19, 180; ‘The Medical and Veterinary Profession – Breach of Etiquette’, *Veterinary Record*, 10 March 1894, 6, 508.

55 ‘M.R.C.V.S.’, ‘The Sister Profession’, *Veterinary News*, 6 September 1913, 426; E. Wallis Hoare, ‘On Getting a Practice’, *Veterinary Record*, 23 November 1901, 14, 332.

56 Gray, ‘Fees’, 792; around 2s. was also a typical consultation fee for 1860s equine practice: see Woods and Matthews, ‘Cow-Leech’, 38; Digby, *Medical Living*, 152–53.

57 Digby, *ibid.*, 6, 77–78.

58 Hancock, *Memoirs*, 63; W. M. Mitchell, ‘The Evolution of Small Animal Practice’, *Veterinary Record*, 14 October 1944, 56, 381–85, 381; Vivash Jones, *Lives*, 244.

59 Alfred Sewell, ‘Experiences of a Veterinary Surgeon’, *Stock-keeper*, 23 December 1898, 582–83, 582.

60 Digby, *Medical Living*, 38.

61 ‘Dogs and Their Doctors—Messrs. C. J. Rotherham and R. H. Singleton, MRCVS’, *LKJ*, 1895, 1, 210–12, 211; ‘Dogs and Their Doctors – Sewell’, 144.

62 ‘Dogs and Their Doctors III – Mr. Alfred Cawdle, MRCVS’, *LKJ*, 1895, 1, 263–65, 265.

63 ‘Dogs and their Doctors – Sewell’, 144; Sewell, ‘Experiences’, 582.

64 ‘Schnapsie’, *LKJ*, 1897, *5*, 283.

65 Alfred Sewell, ‘Kennel Advice’, *Stock-keeper*, 7 January 1898, 7; ‘Parturition’, 247; ‘Kennel Advice’, *IKN*, 8 May 1903, 486.

66 ‘Argand’, ‘Advice’, 180; ‘What We Think’, *Veterinary News*, 3 March 1906, 139–40, ‘The Dog Sanatorium’, advertisement, *IKN*, 14 March 1902, ii.

67 ‘Dogs and their Doctors – Sewell’, 144.

68 Alfred Sewell, ‘My Dogs’, *IKN*, 19 December 1902, 611; ‘Bulldogs’, *IKN*, 25 December 1903, 590.

69 ‘A. H. S.’, ‘Mr A. Cornish-Bowden’, *Kennel Gazette*, April 1921, 126.

70 ‘The Connaught Lodge’, *Kennel Gazette*, December 1907, 554.

71 ‘Initiation of Emil Joachim, George Krehl, Alfred Sewell’, United Grand Lodge of England Freemason Membership Registers, 1751–1921 (hereafter Freemason Registers), Imperial Lodge, Chelsea (no. 1694), 1888–90, 37.

72 ‘Initiation of Frederick Hobday’, Freemason Registers, Kings’ Colonial Lodge (no. 3386), 1915, 266; Vivash Jones, *Lives*, 243; ‘A.H.S.’, ‘Cornish-Bowden’.

73 ‘Initiation of Sir Charles Warren’, Freemason Registers, Quatuor Coronati Lodge (no. 2076), 1884, 172; ‘The Bloodhound Trials’, *Stock-keeper*, 7 October 1898, 297–99, 297.

74 A. J. Sewell, ‘Distemper in Dogs’ (part 1), *Kennel Gazette*, March 1893, 63–64, 63.

75 Sewell, ‘Distemper 1’, 63–64, ‘Distemper in Dogs’ (part 2), *Kennel Gazette*, June 1893, 159–60, G. H. Livesey, ‘Distemper’, *Veterinary Record*, 24 July 1909, 22, 80–84.

76 Worboys *et al**.*, *Modern Dog*, 175.

77 Livesey, ‘Distemper’, 84.

78 Frederick Hobday, *Veterinary Record*, 31 December 1904, 17, 416.

79 M. Ingle-Bepler, ‘Dog Shows and Distemper’, *LKJ*, 1898, 7, 72.

80 Sewell, ‘My Dogs’, 611.

81 Worboys *et al*, *Modern Dog*, 175.

82
*Kennel Gazette*, February 1891, 27.

83 ‘Whispers of Fancy’, *Stock-keeper*, 18 October 1901, 313.

84 ‘Letters of Thanks’, *LKJ*, 1897, 5, 2.

85 Livesey, ‘Experts’, 205.

86 Livesey, ‘Distemper’, 81.

87 Robert Gillard, ‘Distemper and Dog Shows’, *Stock-keeper*, 2 August 1901, 91; G. H. Wooldridge, *Veterinary Record*, 19 March 1910, 22, 618.

88 ‘Inquiry into Alleged Cases of Distemper at Holland Park’, *LKJ*, 1896, 4, 70–74.

89 A. J. Sewell, ‘Distemper at Shows’, *Kennel Gazette*, April 1905, 152.

90
*Ibid*.

91 E. Lionel Stroud, ‘What We Think’, *Veterinary News*, 17 November 1906, 735.

92 ‘Dissemination of Distemper at Shows’, *Kennel Gazette*, February 1913, 70–75, 70; ‘Kennel Club Committee Meetings’, *Kennel Gazette*, March 1913, 125–28, 126.

93 J. H. Bailey at ‘Committee Meetings’, 126.

94 ‘Dissemination of Distemper’, 72.

95 G. H. Livesey, ‘Dog Shows and Quack Medicines’, *Veterinary Record*, 16 February 1907, 19, 535; E. Lionel Stroud, ‘What We Think’, *Veterinary News*, 11 August 1906, 511.

96 ‘The Pathology of Distemper’, *Veterinary Record*, 21 August 1897, 10, 108–09.

97 A. J. Sewell, ‘Scientific Research to Discover a Preventative Against Distemper’, *Stock-keeper*, 31 August 1900, 177.

98 Vivash Jones, *Lives*, 175–82; ‘Scientific Research to Discover a Preventative Against Distemper’, *Stock-keeper*, 13 July 1900, 28.

99 Sewell, ‘Scientific Research’, 177; M. C. Phisalix, ‘Researches on Distemper, *Veterinary Record*, 21 September 1901, 14, 172–73; Henry Gray, ‘Vaccination Against Distemper’, *Veterinary Record*, 18 January 1902, 14, 440–41.

100 Woods, ‘Scientific Experts’, 462; correspondence between A. J. Sewell, J. Woodroffe-Hill and Edwin Brough on ‘Distemper’, *Stock-keeper*, 10 March 1899, 193.

101 ‘Toys - Spaniels and Terriers’, *Stock-keeper*, 7 January 1898, xvii.

102 A. J. Sewell, ‘Phisalix Anti-Distemper Vaccine’, *Veterinary Record*, 13 December 1902, 15, 375–76, 375.

103 A. J. Sewell, ‘Vaccination Against Distemper’, *Stock-keeper*, 2 August 1901, 91; *Stock-keeper*, 8 November 1901, 390.

104 Henry Gray, ‘Abstracts from Foreign Journals’, *Veterinary Record*, 15 April 1899, 11, 601–02.

105 Michael Worboys, ‘Germ Theories of Disease and British Veterinary Medicine 1860–1890’, *Medical History*, 1991, 35, 308–327.

106 Henry Gray, ‘Anti-Distemper Vaccination’, *Veterinary Record*, 29 March 1902, 14, 601–02.

107 Henry Gray, ‘Anti-Distemper Vaccination’, *Veterinary Record*, 6 December 1902, 15, 364–66, 366.

108 A. J. Sewell, ‘Dr. Phisalix’s and Dr. Copeman’s Anti-Distemper Vaccines’, *Veterinary Record*, 22 November 1902, 15, 325–29.

109 Gray, ‘Anti-Distemper Vaccination’, *Veterinary Record*, 6 December 1902, 15, 365–66.

110 A. J. Sewell, ‘Vaccination Against Distemper’, *Kennel Gazette*, February 1903, 51.

111 William Hunting, ‘Vaccination Against Distemper’, *Veterinary Record*, 13 December 1902, 15, 373; Woods, ‘Scientific Experts’, 469.

112 William Hunting, ‘Distemper Vaccine Experiments’, *Veterinary Record*, 25 April 1903, 15, 665.

113
*Kennel Gazette*, May 1903, 167; *Veterinary Record*, 6 June 1903, 15, 765.

114 ‘Report of Committee Formed to Carry out Experiments with the Vaccine of Dr Phisalix for the Prevention of Distemper in Dogs’, *Veterinary Record*, 29 October 1904, 17, 266–69.

115
*Ibid*., 268.

116 ‘The Minority Report of Committee Formed to Carry Out Investigation with the Vaccine of Dr Phisalix’, *Veterinary Record*, 17 December 1903, 16, 380–83.

117 ‘Plain Spoken’, ‘Mr. Gray and the Phisalix Vaccine’, *Veterinary News*, 10 December 1904, 540.

118 Gray remarked that Sewell’s prize bulldog in the distemper hospital ‘would be in a queer street’ if it caught distemper—and Sewell’s bulldog was CALLED ‘Queer Street’; Henry Gray, ‘Anti-Distemper Vaccination and Mr. Sewell’, *Veterinary Record*, 20 December 1902, 15, 400; Sewell, ‘My Dogs’.

119 E. Lionel Stroud, ‘What We Think’, *Veterinary News*, 24 March 1906, 191–93.

120 ‘Election of Council’, *Veterinary News*, 17 June 1905, 279; William Hunting, *Veterinary Record*, 7 April 1906, 18, 695.

121 ‘Cave Canem’, ‘Vaccination’, 714.

122 W. E. Litt, ‘Distemper Vaccine’, *Veterinary Record*, 13 September 1905, 18, 214.

123 Henry Smith, ‘Distemper at Shows’, *Kennel Gazette*, June 1905, 257.

124 Gray, ‘Aspirin and Distemper’, 174.

125 ‘Surgery for Dogs’, *Veterinary News*, 30 September 1905, 474.

126 ‘Kennel Club Committee Meetings’, *Kennel Gazette*, July 1904, 276–83, 277.

127
*Ibid*., 277.

128 ‘General Committee Meeting’, *Kennel Gazette*, May 1905, 207–09, 207.

129 ‘General Committee Meeting’, *Kennel Gazette*, July 1913, 395–400, 398.

130
*Kennel Gazette*, June 1904, 242; General Committee meeting, *Kennel Gazette*, August 1894, 196–97.

131 Derry, *Bred for Perfection*, 70–72.

132 See also account in Worboys *et al.*, *Modern Dog*, 218.

133 ‘The Great Collie Ear Trial’, *Stock-keeper*, 23 December 1898, 587.

134 ‘Whispers of Fancy’, *Stock-keeper*, 16 December 1898, 549.

135 ‘The Great Collie Ear Trial’, *Stock-keeper*, 16 December 1898, 550.

136 ‘The Great Collie Ear Trial’, *Stock-keeper*, 20 January 1899, 42.

137 H. Panmure Gordon, ‘The Great Collie Ear Question’, *Stock-keeper*, 27 January 1899, 65.

138 ‘Collie Ear Trial’, 20 January 1899, 42.

139 ‘The Great Collie Ear Trial’, *Stock-keeper*, 13 January 1899, 23.

140
*Stock-keeper*, 24 March 1899, 224.

141 Thomas Daws, ‘Letter’, *Stock-keeper*, 10 February 1899, 115–16, 116.

142
*Ibid*.

143 W. Charlton, ‘Letter’, *Stock-keeper*, 27 January 1899, 66.

144 Derry, *Bred for Perfection*, 75–76.

145 ‘Kennel Club Committee Meetings’, *Kennel Gazette*, September 1913, 491–510.

146
*Ibid*., 499.

147
*Ibid*., 500.

148
*Ibid.*, 503.

149
*Ibid*., 504.

150
*Ibid*., 501.

151
*Ibid*.

152
*Ibid*., 502–04.

153
*Ibid*., 506.

154 ‘Kennel Club Committee Meetings’, *Kennel Gazette*, February 1914, 81–91.

155
*Ibid*.

156
*Ibid*., 81, 83, 84.

157 ‘Serious Charges against an M.R.C.V.S.—A Dog Case’, *Veterinary Record*, 23 July 1904, 17, 69–71.

158
*Ibid*., 70; Hunting, ‘Distemper Vaccine’.

159 ‘Meeting of Central Veterinary Medical Society’, *Veterinary Record*, 24 February 1906, 18, 600–3, 603.

160 ‘Professional Conduct’, *Veterinary News*, 24 March 1906, 191–93, 192–93.

161 1901 England Census, RG12/85, Folio 123, 3.

162 Livesey, ‘Experts’, 205; Digby, *Medical Living*, 61.

163 ‘Royalty’s Dog-Doctor’, *Veterinary Record*, 3 April 1909, 21, 692.

164
*Ibid*.; ‘Royalty’s Dog Doctor’, *Veterinary News*, 20 March 1909, 141–42.

165 A. J. Sewell, ‘Royalty’s Dog Doctor’, *Veterinary News*, 3 April 1909, 166.

166 Frederick Hobday, ‘Is This Business?’, *Veterinary Record*, 27 May 1905, 17, 754.

167 Valerie Johnson, ‘Some Observations on Animal Nursing’, *Veterinary Record*, 13 January 1962, 73, 71–72; ‘Correspondence’, *Veterinary Record*, 10 April 1909, 21, 700.

168 Hipperson, ‘Entrepreneurs’, 125.

169 ‘Hospital for Animals’, *Veterinary News*, 26 May 1906, 342; ‘An Animals’ Hospital’, *Veterinary Record*, 10 February 1906, 18, 555.

170 Montague Fowler, ‘Animals’ Hospital’, *Veterinary Record*, 26 May 1906, 18, 844.

171 Gardiner, ‘“Dangerous” Women’; Hipperson, ‘Entrepreneurs’.

## Notes

Alison Skipper is a PhD student in the Centre for the History of Science, Technology and Medicine, Department of History, King’s College London and also a practising veterinary surgeon with a long-standing interest in the dog fancy. Her research project, funded by a Wellcome Trust Research Award for Health Professionals, examines the history of understandings of health and disease in pedigree dog breeding from 1890 to the present.

